# TiO_2_ Modified with Organic Acids for the Decomposition of Chlorfenvinphos under the Influence of Visible Light: Activity, Performance, Adsorption, and Kinetics

**DOI:** 10.3390/ma13020289

**Published:** 2020-01-08

**Authors:** Piotr Zawadzki

**Affiliations:** Department of Water Protection, Central Mining Institute, Plac Gwarków 1, 40-166 Katowice, Poland; pzawadzki@gig.eu; Tel.: +48-32-259-2801

**Keywords:** TiO_2_, pyruvic acid, succinic acid, chlorfenvinphos, adsorption, photocatalysis, radicals, scavenger test, modified photocatalysts, kinetics

## Abstract

Photocatalytic decomposition of chlorfenvinphos (CFVP) in the presence of titanium dioxide (TiO_2_) modified with organic acids: pyruvic (PA) and succinic (SA) under the visible light radiation has been studied. The following tests were examined: dose of photocatalysts, adsorption time, pH of the model solution, deactivation of catalysts, the role of oxygen, identification of free radicals for the CFVP decomposition, Langmuir-Hinshelwood kinetics. The synthesized materials were characterized by Scanning Electron Microscopy (SEM) and UV-Vis. At 10 wt.% of acid (90:10) decomposition of chlorfenvinphos was the most effective in the following conditions: dose of catalyst 50.0 mg/L, time of adsorption = 20 min, pH of model solution = 3.0. Under these conditions the order of photocatalyst efficiency has been proposed: TiO_2_/PA/90:10 > TiO_2_/SA/90:10 > TiO_2_ with the removal degree of 85, 72 and 48%. The mathematically calculated half-life at this conditions was 27.0 min and 39.0 min for TiO_2_/PA/90:10 and TiO_2_/SA/90:10 respectively, compared to 98 min for pure TiO_2_. It has been determined that the O_2_^•−^ radicals and holes (h^+^) are the main reactive species involved in the photodegradation of chlorfenvinphos. The results of this study showed that method may be an interesting alternative for the treatment of chlorfenvinphos contaminated wastewater.

## 1. Introduction

The progress of civilization and the increase in the world’s population have made the use of plant protection products in recent years a necessary and comprehensive solution in the elimination of pests in regions poor in food. It is, therefore, an important element of modern agriculture, without which it would be impossible to fight insects, weeds, fungi and other harmful factors for plants. The use of pesticides has brought undoubted benefits to eliminate pests, but taking into account their impact and chronic toxicity, these compounds pose a threat to many living organisms, including humans. Interest in pesticides has focused for many years on four basic properties: selective toxicity, persistence in the environment, bioaccumulation and mobility. The persistence in the environment is probably the most decisive factor when considering the extent of their use. The persistence is often expressed in terms of half-life. The decomposition of pesticides may occur as a result of biological processes as well as chemical and photochemical reactions. The fact that a pesticide loses its characteristic activity does not necessarily mean that it has become a harmless substance. As a result of chemical reactions compounds that are more toxic than primary compounds are often produced [1,2,3].

One of the groups of pesticides belonging to very toxic environmental pollutants are organophosphate insecticides. The organophosphate insecticides are derivatives of phosphoric acid in which the hydroxyl group (-OH) was replaced by the -OR groups derived from alcohol. The organophosphate pesticides inhibit the activity of acetylcholinesterase—one of the most important enzymes for the peripheral and central nervous systems. [(EZ)-2-Chloro-1-(2,4-dichlorophenyl)ethenyl]diethyl phosphate (IUPAC), commonly known as chlorfenvinphos (CFVP), is one of the most important representatives of the organophosphate insecticides family. The CFVP is sold under trade names such as Birlane, Dermatone, and Sapecron. This compound is widely used as a mammalian low toxicity insecticide against pests that destroy potato, rice, carrot, oil seed and maize crops.

The subject pesticide is identified in samples taken from waters around the world [4]. In the United Kingdom, studies focused on monitoring chemicals used for sheep baths were carried out. The study showed that the taken samples of surface water contained such organophosphate pesticides as diazinon, propetamphos, pyrethroids (e.g., cypermethrin, flumethrin) and chlorfenvinphos. The chlorfenvinphos was present at concentrations between 1.0–242.0 ng/L. This compound was also found in groundwater and seawater at around 20.0 ng/L [5,6]. The presence of chlorfenvinphos has also been documented in some surface waters in Poland. According to the results of surface water quality study conducted in 2016 [7], chlorfenvinphos was identified in 32 places at 137 control points in Silesia Region. The pesticide concentration ranged from 0.001–47.4 µg/L. The maximum concentration of the pesticide (47.4 µg/L) was identified in the Wąwolnica stream—intake into the Przemsza River. This state results from the fact that chemicals have been accumulated in the river valley for years as a remnant of the activity of a former chemical plant.

The problem of elimination of substances posing a health hazard for humans and animals has become the reason for the development of new purification technologies, such as the Advanced Oxidation Processes (AOPs) conducted in the presence of titanium (IV) oxide or another semiconductor (a process of photocatalysis). The AOPs methods, although carried out in different reaction variants, have one common chemical feature—the generation of hydroxyl radicals (OH^•^). In the photocatalytic process carried out in the presence of TiO_2_, it is necessary to provide radiation of an appropriate wavelength, carrying energy higher than the bandwidth banned. Titanium dioxide can be activated by light energy with a wavelength λ ≤ 400 nm, so it is necessary to provide expensive lamps emitting ultraviolet radiation in the range of λ = 300–388 nm. Despite the numerous advantages that TiO_2_ has, the attention of engineers and scientists around the world is now directed towards modified photocatalysts. For the practical application of heterogeneous processes involving semiconductors, it is important to increase the efficiency of the visible photocatalysis process, eliminate the agglomeration of TiO_2_ particles, reduce the phenomenon of blocking active sites by intermediate products, and increase the efficiency of separation of catalysts particles from the reaction mixture. Therefore, much attention is currently paid to TiO_2_ modification [8,9,10,11,12]. The above effects can be achieved by using organic acids such as succinic acid or pyruvic acid. In this study, succinic and pyruvic acid were selected for modification of commercial TiO_2_. Pyruvic and succinic acids are naturally available acids (Krebs Cycle Compounds). Pyruvic acid is the simplest of the alpha-keto acids and succinic acid is a dicarboxylic acid. They are characterized by low toxicity towards living organisms. For example, the EC_50_ after 48 h relative to Daphnia magna is 374.2 mg/L (succinic acid), while the LD_50_ relative to mice is 3.5 g/kg (pyruvic acid). The amounts used during the study were several hundred times smaller. Modification with these organic acids is a simple and fast technology of modification [13,14], because carboxyl groups (-COOH) from pyruvic acid and carboxyl and ketone (C=O) groups from succinic acid can form bonds with metal oxide nanoparticles and play a positive role in extending the wavelength response range [15,16]. The use of succinic and pyruvic acid ensures higher photocatalytic activity of the catalysts by forming new absorption bands (waves above 400 nm), i.e., in the visible light range. This is due to the transfer of electrons from PA/SA to the conduction band of TiO_2_ and the appearance of a new C-O-Ti bond [17]. The role of acids is also to improve adsorption of pollutants by increasing the specific surface area, to reduce the phenomenon of the TiO_2_ agglomerate formation and to inhibit the anatase-rutile transformation [18,19]. According to literature data [20], thermal treatment of semiconductors leads to the formation of materials with a lower specific surface area due to agglomeration of nanoparticles of catalysts. The use of acids reduces this phenomenon, leads to an increase in crystallites and thus an increase in the active surface of the catalysts.

The literature review shows that the decomposition process conducted in the presence of titanium (IV) oxide is an efficient and effective method of pollutants degradation but poor reuse of TiO_2_, energy-consuming UV lamps and long photooxidation time are required. The novelty of the presented study, in comparison to other methods described in the literature, is the possibility of TiO_2_ activation with low energy-consuming Vis lamp due to the use of succinic and pyruvic acids as photocatalyst modifiers, greater adsorption of chlorfenvinphos, photooxidation over a wide pH range, possibility of reuse of catalyst.

In order to remove pollutants that show low biodegradability and high toxicity, it is economically justified to applied method as presented in this study. This method turns out to be an interesting alternative to other treatment techniques.

The aim of the study was to examine the possibility of decomposition of chlorfenvinphos in the presence of photocatalysts modified with organic acids (pyruvic and succinic) under visible light radiation.

## 2. Materials and Methods

### 2.1. Materials

The chlorfenvinphos PESTANAL^®^ with a purity of >95% was purchased from Sigma-Aldrich (Poznań, Poland). Physico-chemical characteristics of the compound is presented in Table 1. Modified catalysts obtained on the basis of titanium (IV) oxide (Sigma-Aldrich, Poznań, Poland) were used as catalysts for the pesticide decomposition process. The physico-chemical characteristics of commercial TiO_2_ are summarized in Table 2. All chemicals were of analytical grade and the highest purity available.

### 2.2. Model Solution

Model solutions were prepared on the basis of deionized water with the addition of the analytical standard—chlorfenvinphos (CFVP) PESTANAL^®^. Solutions were prepared by diluting the chlorfenvinphos analytical standard in deionized water. The conductivity of model solutions was 0.196 mS/cm. To maintain a constant ionic strength, the model aqueous solutions contained 0.01 mol/L of NaNO_3_. The solutions were stored at 4 °C in glass flasks, this ensured no adsorption of the pesticide particles on the walls of the vessel. In order to maintain satisfactory accuracy and repeatability of instrumental analysis, the initial concentration of the aqueous solution for all experiments was 1.0 mg/L. The pH of the model solution before and after addition of the compound was 6.0. The pH of the solution was monitored using the Elmetron CPC-511 pH-meter (Zabrze, Poland).

### 2.3. Instrumental Analysis

The analytes contained in liquid samples before and after the advanced oxidation process were subjected to the HPLC chromatographic analysis with the UV detector model 1200 from Perlan Technologies (Warsaw, Poland) in accordance with PN-EN ISO 11369:2002 standard [21]. The analysis was preceded by the separation of the compound using the solid-phase extraction (SPE). Prior to the extraction, the particles of photocatalysts were separated from the samples using a filtration apparatus through a 0.45 μm filter made of cellulose acetate (Microlab Scientific Co., Ltd., USA). The filtration did not affect the retention of the tested compound on the filter. The extraction process was carried out on the CHROMABOND^®^ C_18_ ec columns. The extraction column was filled with a non-polar C18 sorbent (capacity of 6.0 mL, the adsorbent mass of 500.0 mg). The column bed was conditioned with methanol (5.0 mL) and deionized water (5.0 mL). Samples were dosed at flow rate of 5 mL/min. Then the columns were dried, in the first phase with air, then with inert gas (nitrogen). The compounds adsorbed on the bed were eluted with 1.0 mL of methanol with a 15 min retention time, then they were eluted with another 1.0 mL of methanol. The process was completed by concentrating the compound in vacuo with inert gas (nitrogen).

The extract was analysed using the HPLC liquid chromatograph (UV detector, λ = 218 nm). The device was equipped with the Zorbax^®^ SB-C18 packed column with dimensions of 15.0 cm × 4.6 mm × 5.0 µm.

### 2.4. Synthesis of Photocatalyst Samples

Modification of the photocatalysts is essentially aimed at obtaining active materials in visible light (λ > 400 nm), and thus increasing the ability to absorb visible radiation. Another application of modifications (such as e.g., transition metals, ultrasound, activated carbon, polymers, elemental carbon, nitrogen, sulphur) is to increase adsorption of the pollutants, neutralization of intermediate decomposition products and better separation of TiO_2_ suspension from the liquid phase after the process [22,23].

As part of the study, the commercial titanium (IV) oxide and two types of catalysts, consisting of titanium (IV) oxide and pyruvic or succinic acid, were evaluated. Each of the materials has been given an appropriate symbol (Table 3). The modified photocatalysts were obtained by the wet impregnation method, preparing a suspension of titanium dioxide in deionized water with the addition of an appropriate amount of acid. The mixture was shaken vigorously for 30 min in the dark, at room temperature, then dried at 100 °C for 16 h. Five pyruvic acid-modified photocatalysts and five succinic acid-modified photocatalysts were prepared. The photocatalysts contained various acid concentrations, i.e., 99:1; 90:10; 80:20; 50:50 and 20:80 (*w*/*w*). The dried catalysts were washed with distilled water to remove unbound acid and dried again at 100 °C for 16 h.

### 2.5. Experimental Procedures

#### 2.5.1. Characterization of Photocatalysts Samples

The photocatalytic activity of each sample was determined by examining the degree of decomposition of chlorfenvinphos and conducting an analysis of the photocatalytic kinetics. The photocatalysts were examined by the diffuse reflection spectroscopy (UV-DRS) and the scanning electron microscopy (SEM). The UV-Vis photocatalysts absorption spectra were measured using the V-750 spectrophotometer (Cracow, Poland) made by Jasco. The morphology and structure of semiconductors were examined using the Hitachi SU-3500N electron microscope (SEM, Krefeld, Germany). The catalyst samples were applied to a graphite adhesive plaster and placed on a microscope (variable vacuum).

#### 2.5.2. Effect of Different Dose of Catalysts

**Figure 1:**
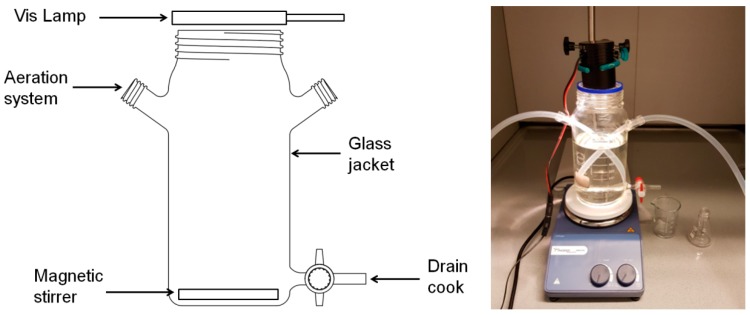
Scheme of conducting the chlorfenvinphos decomposition process under the influence of visible light.

Studies on the selection of the optimal dose of the photocatalysts were carried out at room temperature and ambient pressure in a glass reactor of 500.0 mL volume. The ionic strength of the solution was maintained with 0.01 mol/L of NaNO_3_. The mixture containing pesticide at a concentration of 1.0 mg/L was placed on a magnetic stirrer and then mixed with the addition of selected catalysts in the dose range from 10.0 to 125.0 mg/L. The selected dose range is smaller than the optimal doses presented in the literature [24,25], while from the economic point of view, reduction of the amount of catalyst should be considered. For example, in the research of Garg et al. [26] the decomposition of bisphenol A was studied in the dose range from 20 to 175 mg/L. It should also be remembered that the intention of this study was to determine the basic operating parameters of the reaction system, so the tests were carried out under “ideal” conditions, e.g., without the influence of inorganic substances or other organic substances. Pure titanium (IV) oxide and two randomly selected modified semiconductors: TiO_2_/PA/99:1 and TiO_2_/SA/80:20 were used for the study. The source of Vis radiation was switched on immediately after the catalysts were introduced into the reaction mixture, therefore the results obtained in this step do not take into account the degree of the compound adsorption. The reaction mixture was irradiated with Vis continuously for 60 min. After that time, samples were taken for analysis. The 10 W tungsten lamp QTH10/M from Thorlabs Inc. (Newton, NJ, USA), located above the reaction vessel (Figure 1), was used for exposure. The lamp emits radiation with a wavelength λ = 400–2200 nm, but for the purpose of the study the FGS900M filter, mounted using a cage filter wheel (model LCFW5) system from Thorlabs Inc., was used to cut off the radiation spectrum bands above 710 nm. Thus the lamp emitted radiation in the visible light range (λ = 400–710 nm). The photodegradation process was carried out without adding an external oxygen source comes from an aeration pump. The only source of oxygen was water, in wich the process was conducted. The impact of the presence of external oxygen source on the efficiency of the compound decomposition is discussed in Section 2.5.7.

#### 2.5.3. Optimal Modification of the Catalysts

Based on the decomposition of chlorfenvinphos in the presence of pure TiO_2_ and the photocatalysts modified with organic acids, as a result of irradiation of the model solutions with visible radiation, the catalysts were selected and further tested. The dose of photocatalysts was 50.0 mg/L. Tests were carried out under the same conditions as described in Section 2.5.2. The only change was the sampling times for testing. Irradiation was carried out continuously for 60 min, with samples for testing taken after 5, 10, 15, 30 and 60 min by means of a drain cook, which is an integral part of the reactor. The photodegradation process was carried out without oxygen present in the reaction system.

#### 2.5.4. Optimal Adsorption Time

Determining the contact time of the adsorbate (CFVP) with the adsorbent (catalyst) is an important issue from the point of view of the process of pollutants removing. The initial volume under the study was 500.0 mL. An amount of pesticide was added to the model solution so that the final concentration of adsorbate was 1.0 mg/L. The dose of adsorbents was 50.0 mg/L. During the experiment, no aeration pump (oxygen source) was used. Sorption of pollutants proceeded in the dark. The test solution was placed on a magnetic stirrer for 30 min continuously, and samples were taken after 1, 3, 5, 8, 10, 15, 20, 25 and 30 min.

#### 2.5.5. Effect of pH

As part of the experiments, the effect of the initial pH of the solution on the efficiency of decomposition of chlorfenvinphos was investigated. The following pH values were tested: pH = 3.0; pH = 6.0 and pH = 9.0. The pH was corrected with 0.1 mol/L of HCl or 0.2 mol/L of NaOH obtained from Sigma-Aldrich. The change in pH was monitored using the CPC-511 pH-meter from Elmetron (Zabrze, Poland). The model solution with a concentration of 1.0 mg/L was added before the pH was corrected. Then, after reaching the desired pH, appropriate amount of catalysts were added to the solution to the final dose of 50.0 mg/L. Before switching the radiation source, the 20-min contact time of the catalysts with the model solution has been provided (adsorption). Sorption of the pollutants proceeded in dark, then the Vis lamp was switched on. The study was carried out continuously for 60 min, with samples for chromatographic analysis taken after 5, 10, 15 and 30 and 60 min of the process duration. The photodegradation process did not take place with participation of oxygen supplied to the reaction system.

#### 2.5.6. Deactivation Tests

Deactivation of the photocatalysts was carried out under similar conditions to those described in the previous sections. The pH of the model solution was 6.0. Studies on the determination of materials viability were carried out based on 5 cycles of the chlorfenvinphos decomposition at a constant catalyst concentration. The decomposition degree of the compound was determined after each cycle of the conducted decomposition. The duration of the photodegradation process was 60 min. After this time, samples were taken for analysis. Then another cycle of irradiation of the mixture was started, preceded by separation of the catalyst particles. The photocatalysts particles were recovered by filtering the reaction suspension through a filtration apparatus, equipped with a 0.45 µm membrane filter made of cellulose acetate (Microlab Scientific Co., Ltd.). The reaction vessel was flushed and the contents of the vessel were subjected to filtration. As part of preliminary tests, it was determined that the recovery rate of the catalyst particles was >99.9%. The photodegradation process did not take place in the presence of oxygen supplied to the reaction system.

#### 2.5.7. Influence of Oxygen

In order to determine the effect of dissolved oxygen on the efficiency of the chlorfenvinphos photocatalytic oxidation, the tests were carried out comparatively, with and without the addition of oxygen. The research procedure was similarly to the previous section. The photodegradation time was set to 60 min and after that time the samples were taken. An aeration pump (Miniboost 200, Aquael Company, Warsaw, Poland) with a capacity of 2 × 100 L/h was used as a source of oxygen.

#### 2.5.8. Radical Scavenger Test

The radical scavenger test was used to determine the main radical species involved in the degradation of chlorfenvinphos. Scavengers inhibit the free radicals or deactivate them, thus preventing them from reaction with the compounds present in water. The testing procedure was carried out similarly to the previous studies. The ethylenediamine tetraacetate (EDTA-2Na) of 98.5% purity, hydroquinone of ≥ 99.5% purity and methanol (MeOH) of 99.8% purity from Sigma-Aldrich were added to the test samples before adding the catalysts. The scavengers were always used in concentration of D_scav._ = 50.0 µM.

All the experiments described in Section 2.5.2, Section 2.5.3, Section 2.5.4, Section 2.5.5, Section 2.5.6, Section 2.5.7 and Section 2.5.8 were carried out independently in triplicate. The data presented in the next sections include the average values.

### 2.6. Kinetics

Many studies suggest that the oxidation rate of organic substances fit with the Langmuir-Hinshelwood (L-H) kinetics model [27,28]. Under ideal conditions, the L-H model can be expressed by Equation (1). Based on this equation, the kinetics of the chlorfenvinphos decomposition was analysed and the following pseudo first-order reaction parameters were determined: reaction rate constant *k*, determination coefficient *R*^2^, and half-life *t/2*.

(1) $$r=-\frac{dc}{dt}=\frac{k\times KC}{1+KC}$$

where:

*r*—oxidation rate of pollutants, mg min/L
*C*—concentration of pollutants, mg/L
*k*—reaction rate constant, min^−1^
*K*—constant balance
*t*—contact time, min

## 3. Results of Tests and Their Discussion

### 3.1. UV-DRS and SEM Test

**Figure 2:**
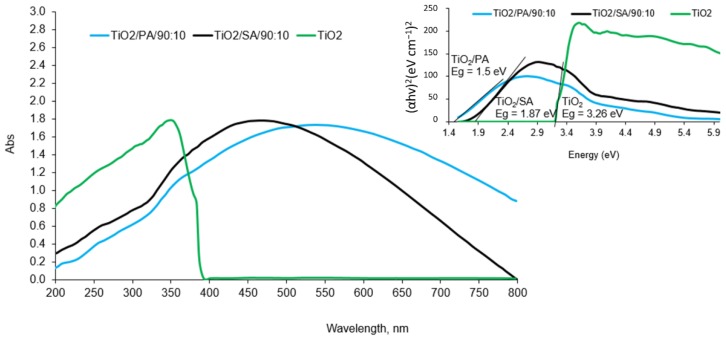
The UV-Vis spectra of the tested photocatalysts.

The modification changed the optical properties of the commercial titanium (IV) oxide. The results are shown in Figure 2. The commercial TiO_2_ shows an absorption edge at approx. 380 nm, while the modification of the photocatalysts shifted the absorption of light towards the visible light. Compared to the pure TiO_2_ a shift was observed in the absorption maximum to a wavelength equal to λ = 468 nm for TiO_2_/SA/90:10 and *λ* = 528 nm in the case of a semiconductor marked with a symbol TiO_2_/PA/90:10. The band gap energy was calculated on the basis of [29]. Samples modified with organic acids did not show a sharp absorption edge as in the case of the pure TiO_2_ and were characterized by having a “tail” reaching about 800 nm. The appearance of new absorption spectra may indicate changes in the structure of TiO_2_ modified with organic acids, that were caused by reaction between the groups present on the surface of titanium dioxide and the products of acid decomposition [13].

**Figure 3:**
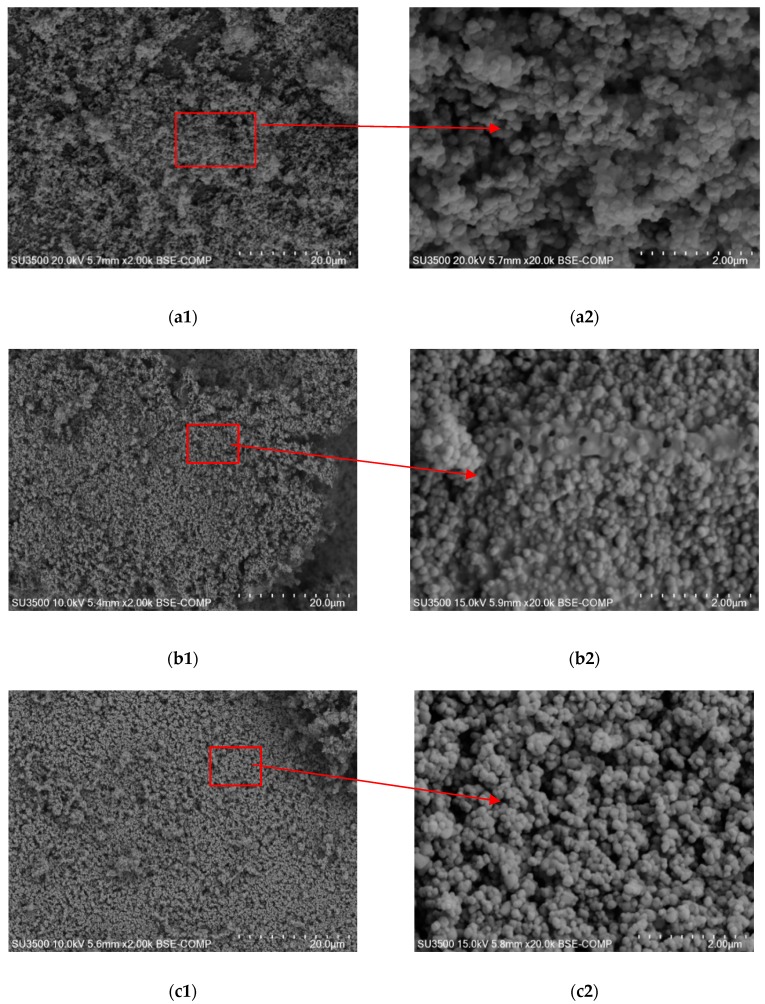
Comparison of SEM images for he pure TiO_2_ (**a1**,**a2**) and the catalysts modified with pyruvic acid (**b1**,**b2**) and succinic acid (**c1**,**c2**) in proportions 90:10.

Figure 3 shows micrographs obtained for the pure TiO_2_ and titanium dioxide modified with pyruvic (90:10) and succinic (90:10) acid. The pure titanium (IV) oxide has a homogeneous, regular, spherical shape with a single particle size below 100 nm. Modified TiO_2_ surface study showed that the modification of the photocatalyst does not significantly affect the shape of titanium (IV) oxide nanoparticles in comparison to the pure TiO_2_ particles. However, after modification with acid, the shape of the new semiconductor slightly lengthened, which could have contributed to the higher photocatalytic activity of the TiO_2_/PA and TiO_2_/SA particles in comparison to pure TiO_2_ [30]. In addition, in the synthesized catalysts the TiO_2_ particles were not found as complex of agglomerates as in the case of a commercial semiconductor. The dispersion of particles increases the specific surface area, which improves the photocatalytic properties of the catalysts labelled as TiO_2_/PA/90:10 and TiO_2_/SA/90:10 [31]. Studies shows that the increase in the specific surface area of the catalysts as a result of acid interaction may result from a change in the size of TiO_2_ crystallites. Catalysts with smaller crystal sizes have a larger specific surface area, which increases the adsorption of pollutants and ensures higher photocatalytic activity. The study [32] showed that catalysts with smaller crystallite sizes have a larger surface area. Research conducted by Mair et al. [33] showed that a smaller size of crystallites affects a higher degree of adsorption and mineralization through a more of edges and corner sites for the formation of TiO^3+^ centers where O^2−^ radicals are formed. Apparently, the addition of acids increased the specific surface area by reducing particle agglomeration and reducing TiO_2_ crystals. This in turn can affect the degree of interaction between the photocatalyst and organic pollutants.

**Figure 4:**
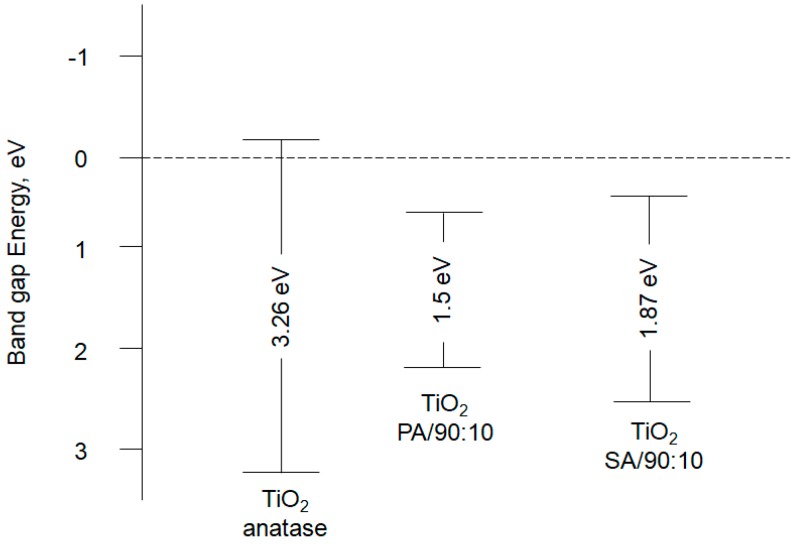
Valence and conduction bands of the tested photocatalysts.

The values of the band gap energy and the location of valence and conduction bands are shown in Figure 4. The band gap energy was calculated according to [29]. The energy of the conduction band was calculated according to the formula E_CB_ = E_VB_ − E_g_, while the energy of the valence band according to the formula E_VB_ = 1.46 + 0.5E_g_ on the base of [34,35]. Due to the reduction of the band gap energy of the modified photocatalysts, the transfer of electrons from the valence band (VB) to the conductivity band (CB) is facilitated. This phenomenon has been commented in Section 3.8.

### 3.2. Effect of Catalyst Dosage

Application of an optimal dose of a photocatalyst makes the decomposition process to be carried out in the most effective way. The photocatalyst dose limit value was determined based on the initial concentration of chlorfenvinphos removed from solution as well as the operating conditions and geometry of the reactor in which the decomposition was carried out.

**Figure 5:**
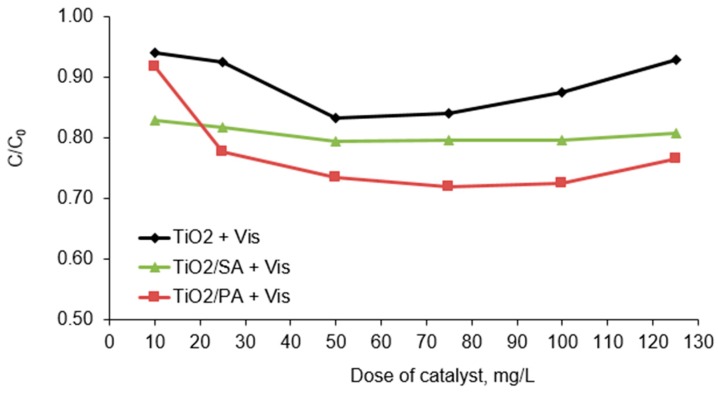
Decomposition degree of chlorfenvinphos in the presence of the selected catalysts. Conditions of experiment: C_0_ = 1.0 mg/L; V_r_ = 500.0 mL; t_ir_ = 60.0 min; pH = 6.0.

The optimal dose of the catalysts used during the tests was determined experimentally. Therefore, the commercial titanium (IV) oxide and the selected photocatalysts doped with different amounts of acids (TiO_2_/PA/99:1 and TiO_2_/SA/80:20) and in the doses ranging from 10.0 to 125.0 mg/L were introduced into the model solution and irradiated with Vis. The presented results do not include adsorption of the compound. The phenomenon of the CFVP adsorption was investigated in Section 3.4. As shown in Figure 5, the dose of the catalyst had an impact on chlorfenvinphos degradation. In the low dose range of the tested materials (from 10.0 to 50.0 mg/L), an increase in the decomposition efficiency of the tested compound was observed. In turn, at higher doses (from 75.0 to 125.0 mg/L) the increase in decomposition compared to the dose of 50.0 mg/L was insignificant or lower. Therefore, it was determined that the decomposition of model compound occurs with the highest efficiency at the dose of 50.0 mg/L of the photocatalysts. Despite the fact that CFVP decomposition was higher in the presence of the dose of 75 mg/L than 50 mg/L, the difference between this efficiency was insignificant (~1%). In addition, economic aspects were also taken account. Slight differences in the CFVP removal and the risk of faster sedimentation of the catalysts determined the choice of the 50 mg/L dose. That dose was chosen as optimal for further study. The increase in efficiency of the chlorfenvinphos decomposition along with the increase in the catalysts doses can be attributed to the adsorption of the compound on the surface of the catalysts and inside its pores, as well as the generation of more free radicals, that leads to a higher degradation of the pollutant. Whereas the reduction in the photodegradation efficiency can be explained by faster sedimentation of the catalyst particles to the bottom of the reactor, the effect of radiation shielding, and the effect of screening of the excessive amount of particles. Particular importance is attached to the shielding effect, which results from the negative impact of pollutants present in the solution, and a high dose of the photocatalyst. In the study we probably deal with the second case, i.e., the increase in turbidity of the solution, caused by too high dose of catalysts, limits the possibility of radiation reaching the surface of the catalyst. These phenomena depend on the geometry of the reactor and the operating conditions of the system. The similar results were noted by the authors of the works [24,36,37,38]. However, as part of this research stage, it can be stated that the modification of commercial TiO_2_ with organic acids brings satisfactory results. First of all, the addition of acids, in particular succinic acid, caused that the degree of the CFVP photodegradation was not only higher but was similar in almost whole range of the tested doses. This may indicate a positive effect of succinic acid on the elimination of radiation shielding phenomenon.

### 3.3. Modification of the Catalyst

**Figure 6:**
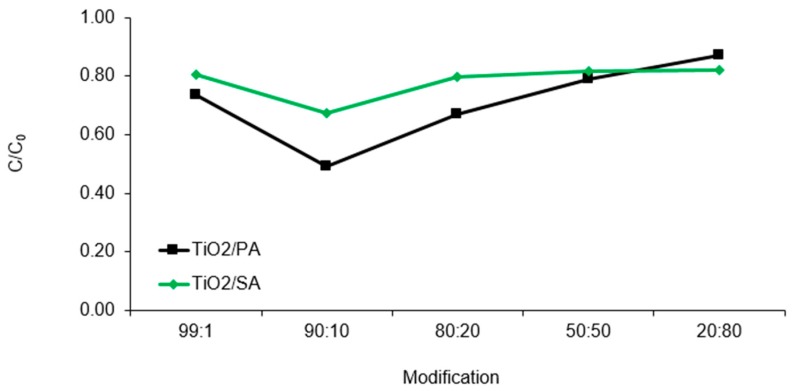
Chlorfenvinphos decomposition in the presence of various modifications of photocatalysts. Condition of experiment: C_0_ = 1.0 mg/L; D_c_ = 50.0 mg/L; V_r_ = 500.0 mL; t_ir_ = 60.0 min; pH = 6.0.

The results of the conducted study, on the basis of which the optimal modification of the photocatalysts was selected, are shown in Figure 6. The presented results do not take into account the adsorption process. The phenomenon of the CFVP adsorption was studied in Section 3.4. Among the tested pyruvic and succinic acid-modified catalysts, the highest efficiency was observed for the materials mixed in proportions 90:10. For the pyruvic acid-modified catalyst, the degree of the chlorfenvinphos decomposition was 51.0% after 60.0 min of the reaction. The use of larger amounts of acid reduced the efficiency of the compound decomposition. For example, for the catalyst labelled as 20:80, i.e., the highest of the tested acid content, the degree of decomposition decreased to 13.0% after 60 min of the photodegradation process. A similar phenomenon was observed for the succinic acid-modified catalyst. Increasing the share of acid in the total mass of the catalyst caused a reduction of the efficiency of the chlorfenvinphos degradation, from 33.0% for the catalyst with the symbol 90:10 to about 20.0% for materials with a higher participation of acid.

The photodegradation processes catalysed by TiO_2_/PA/90:10 and TiO_2_/SA/90:10 proceeded more intensively compared to the other tested materials, as evidenced by the kinetic parameters presented in Table 4. The kinetics of the conducted processes indicated that the reaction rate constant *k* for the TiO_2_/PA/90:10 catalyst is more than three times higher than the value of *k* determined for other pyruvic acid-modified catalysts. Also for the TiO_2_/SA/90:10 catalyst the reaction rate constant was twice as high compared to other modifications carried out with succinic acid. The shortest half-life *t/2* was observed for the material with the symbol TiO_2_/PA/90:10 (63 min). It also turned out that the use of too much of the pyruvic acid in the TiO_2_/PA/20:80 catalyst resulted in elongation of the decomposition half-life to 315.0 min, and it was longer time compared to the pure titanium (IV) oxide.

Although it was found higher CFVP decomposition compared to the pure TiO_2_, the increase in the amount of acid contributed to the decrease in efficiency of the pollutant decomposition. The decrease in the photocatalytic activity of the catalysts could have been associated with the blocking of active sites on the photocatalyst surface, therefore the particles of CFVP could not attach to them and undergo decomposition. In addition, higher amounts of acids might cause a weaker shift of the absorption band in the visible light compared to the optimal dopant of acid. However, at the smallest proportion (99:1) there could also be no shift of the absorption band in the visible light, which for the pure TiO_2_ is λ < 400 nm. It is also worth adding that during preparation procedure the pyruvic acid-modified catalysts changed colour from white to yellow, while those modified with succinic acid changed into dark orange. The sensibilization effect could also contribute to higher activity of the modified semiconductors. The color change of the photocatalysts may results from the structure of titanium (IV) oxide. It is characterized by nearly 40% of incompletely coordinated Ti atoms that can accept two lone electron pairs from electron donors (succinic or pyruvic acids). The color change of titanium dioxide can be attributed to the charge transfer from PA/SA to TiO_2_ as a result of which the PA-TiO_2_ or SA-TiO_2_ complex is formed. The sensibilization of catalysts by means of other admixtures was shown in the studies [29,39,40].

### 3.4. Optimal Adsorption Time

**Figure 7:**
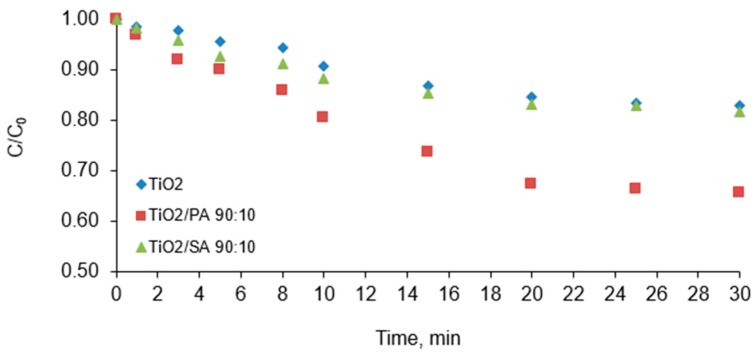
Effect of the contact time on the degree of chlorfenvinphos adsorption. Condition of experiment: C_0_ = 1.0 mg/L; D_c_ = 50.0 mg/L; V_r_ = 500.0 mL; t_sor_ = 30.0 min; pH = 6.0.

In the next step the optimal sorption time was determined. It is important to consider the contact time from the point of view of the proper distribution of the catalyst in the whole volume of the solution and ensuring the highest degree of adsorption of the contaminant before starting the photodegradation process. It was observed that in the case of the tested photocatalysts the CFVP adsorption takes place in the first 20 min of the experiment (Figure 7). This time is needed to reach the adsorption-desorption balance. After 20 min of contact time, the achieved degree of removal was 16.0%, 33.0%, and 17.0% respectively for the TiO_2_, TiO_2_/PA/90:10 and TiO_2_/SA/90:10. In the following minutes, the CFVP adsorption was not significant. The degree of compound removal was the highest after 20 min of time, but already in the first minute about 2% removal of chlorfenvinphos was obtained, which is related to the physical properties of the tested materials, i.e., the effect of their specific surface area, which for the commercial TiO_2_ is about 50 ± 15.0 m^2^/g. For the pyruvic acid-modified TiO_2_, the degree of CFVP removal was twice as high as for the pure TiO_2_ and succinic acid-modified TiO_2_. Chemical modification of inorganic titanium (IV) oxide particles using the organic particles, is of particular importance and causes stabilization of the TiO_2_ nanoparticles, prevents agglomeration of the catalyst particles and improves the surface properties. The results suggest that the specific surface area of the catalyst may have been increased by pyruvic acid, resulting in an increase in the amount of chlorfenvinphos adsorbed by this material. The increase in the specific surface area is important in terms of the amount of active sites of TiO_2_ on which its photocatalytic activity depends. As the surface increases, the amount of adsorbed pollutants and the amount of free radicals increases. Similar conclusions were drawn in studies [32,39,41].

### 3.5. Effect of pH on the Chlorfenvinphos Degradation

**Figure 8:**
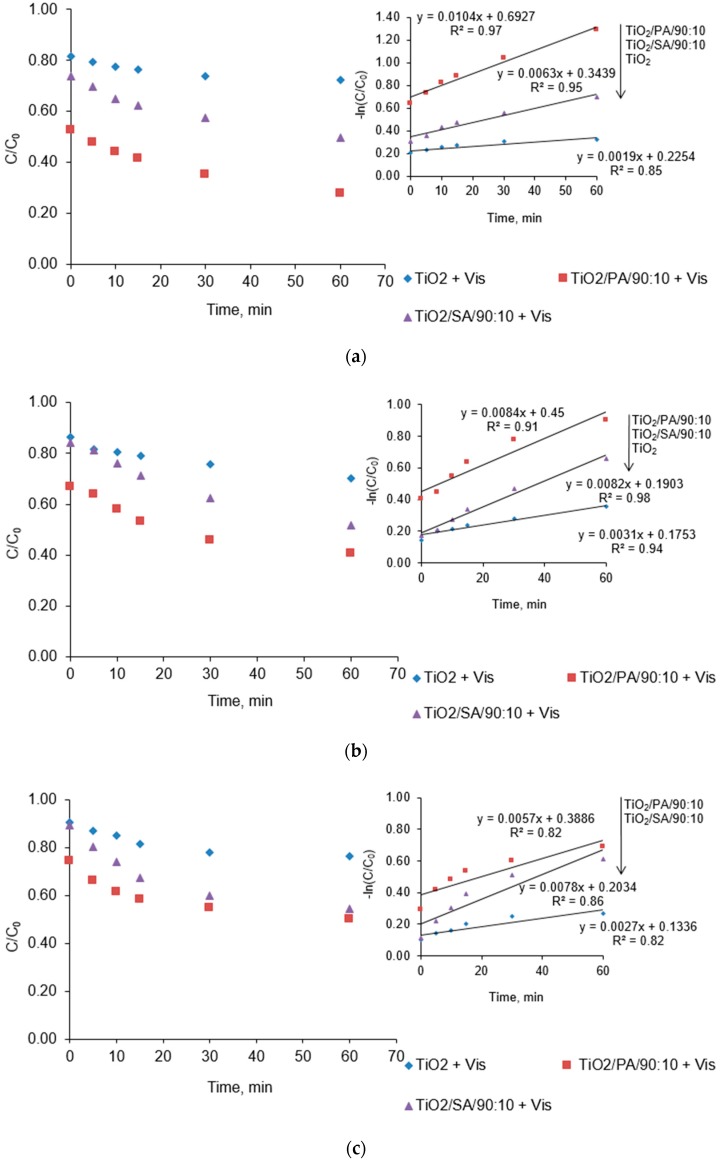
Reduction in the initial concentration of CFVP in the model solution at (**a**) pH = 3.0, (**b**) pH = 6.0, (**c**) pH = 9.0. Conditions of experiment: C_0_ = 1.0 mg/L; D_c_ = 50.0 mg/L; V_r_ = 500.0 mL; t_sor_ = 20.0 min; t_ir_ = 60.0 min.

The decomposition of chlorfenvinphos catalysed by the selected semiconductors was tested in the pH range from 3.0 to 9.0 for 60 min. As shown in Figure 8, the change in the initial pH of the solution affected on the CFVP decomposition efficiency. After 60 min of irradiation at the pH = 6.0, the 30% decomposition degree of CFVP in the process catalysed by pure TiO_2_ was found. This value was the highest compared to other pH tested and was associated with the value of semiconductor isoelectric point (pH_pzc_). For the titanium dioxide particles, the value of pH_pzc_ is in the range from 6.0 to 6.5, on average about 6.25 [42,43]. This is due to the effect of pH on the surface charge of TiO_2_. In an alkaline environment (pH > 6.0) the surface of a pure semiconductor is positively charged, while in an acidic (pH < 6.0) it is negatively charged. After 60 min of irradiation the concentration of CFVP was reduced by 24% at pH = 9.0, while the lowest degree of the CFVP decomposition was observed at the lowest of the tested pH, i.e., pH = 3.0 (28.0%). However, the change in the pH of the model solution had the greatest effect on the adsorption of the compound. The degree of the CFVP adsorption at pH = 3.0 was the highest in each of the tested catalysts. For example, adsorption of the compound by pyruvic acid-modified semiconductor at pH = 3.0 was 47% compared to 25% at pH = 9.0. In the presence of the catalyst labelled as TiO_2_/SA/90:10 the change in pH of the model solution did not have a significant impact on the final chlorfenvinphos decomposition. After 60 min of the photocatalysis, the CFVP decomposition was about 50%. Process carried out in the presence of TiO_2_/SA/90:10 remained active over a wide pH range, suggesting it has low sensitivity to pH changes. The analysis of photocatalytic oxidation kinetics also demonstrated similarity in this case (Table 5). The reaction rate constant was between *k* = 0.0063 and *k* = 0.0082 min^−1^, while the half-life was between 85 and 102 min. In turn, the highest degree of the CFVP decomposition was observed in the pH = 3.0, to which the pyruvic acid-modified catalyst was added. After 60 min of irradiation, the calculated decomposition degree was 72%, and it was a value of 12% and 22% higher for pH = 6.0 and pH = 9.0 respectively. The mathematically calculated half-life was determined at *t/2* = 67.0 min. Due to the high adsorption potential of the TiO_2_/PA/90:10 semiconductor, tested compound was more degraded as a results the generated free radicals. The change in pH of the model solution directly affects the photocatalyst surface charge, its hydrophobic properties and the amount of generated radicals [44]. The change in pH also affects the change in the micropollutants electric charge, thus resulting in a change in their susceptibility to adsorption on the catalyst surface. The CFVP dissociation constant *pK_a_* has not been determined, but based on the analysis of dissociation constants of other organophosphorus pesticides it can be concluded that this pesticide is negatively charged at pH > 6, therefore it is better adsorbed at lower pH. For organic pollutants (e.g., azo dyes), similar conclusions were made by Alkaim et al. (2014) [45]. At pH <6, strong dye adsorption was observed on TiO_2_ particles as a result of electrostatic attraction of positively charged TiO_2_. At pH >6.8, negatively charged dye particles are repelled. Modification of the TiO_2_ surface with pyruvic acid could cause change in the surface properties of titanium (IV) oxide, hence the high degree of chlorfenvinphos decomposition resulted from the generation of more oxidizing radicals. A similar explanation was proposed by Fernandez-Domene et al. (2019) [46].

### 3.6. Lifetime of the Catalysts

Pure TiO_2_, TiO_2_/PA/90:10 and TiO_2_/SA/90:10 catalyst were used to determine the possibility of photocatalysts reuse. The experiment was carried out in five decomposition cycles of chlorfenvinphos. After the first cycle, the catalysts were separated from the solution and used for further cycles. Easier separation of the modified catalysts from the solution compared to pure TiO_2_ has been found. For pure TiO_2_ the process of filtering the reaction slurry required five times flushing, while for the modified TiO_2_ the reactor was flushed three times. The particles of modified catalysts sedimented after about 10 min, while the commercial titanium (IV) oxide particles after about 30 min. The similar separation tests were conducted by Liu et al. (2007) [47] in which the TiO_2_ modified with activated carbon sedimented after 5 min, while the pure titanium dioxide after 20 min. Despite faster sedimentation of modified photocatalysts, their performance was still higher than in commercial TiO_2_.

**Figure 9:**
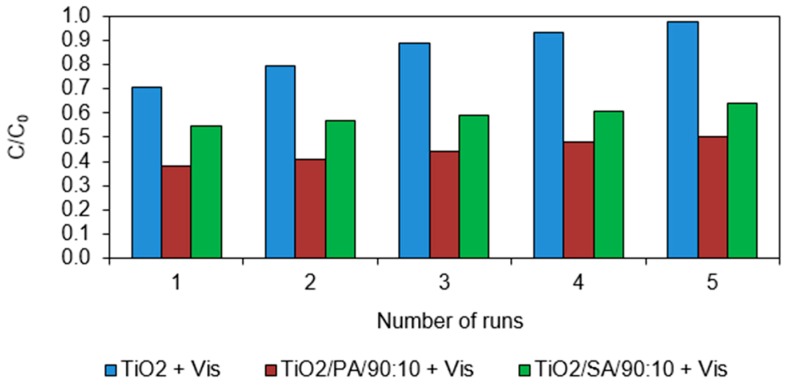
Effect of the number of cycles on the CFVP decomposition on the tested catalysts. Conditions of experiment: C_0_ = 1.0 mg/L; D_c_ = 50.0 mg/L; V_r_ = 500.0 mL; t_sor_ = 20.0 min; t_ir_ = 60.0 min.

The results presented in Figure 9 show that activity of pure TiO_2_ during 5 cycles decreased from 27% to 2% (reduction of efficiency by 25%). However, the decomposition of CFVP in the presence of TiO_2_/PA/90:10 decreased after 5 cycles by 12%, while the decomposition efficiency was still high (50%) after the fifth cycle. The decomposition of chlorfenvinphos catalysed by TiO_2_/SA/90:10 reached value of 36% after the fifth cycle, while the decomposition degree decreased by 10% in comparison to the first cycle. The results of this experiment are extremely important from the practical point of reuse of the materials for pollutants removing from water and wastewater, especially from the economic point of view. The phenomenon of reducing the efficiency of the CFVP decomposition on commercial TiO_2_ is associated with the blocking of active sites on the catalyst surface by the intermediate decomposition products, formed during the process. It is a competitive process in relation to the decomposition of compound, resulting in a reduction of the activity of the catalysts. The modifications of the commercial TiO_2_ probably reduced the phenomenon of formation of the TiO_2_ agglomerates, so there was no active surface reduction effect, and thus it was possible to generate more free radicals. In addition, the organic acids used in the study are electron acceptors, which prevents recombination of electron-hole pairs [48]. The results indicate that the proposed method creates promising possibilities for the reuse and applied in practice for the degradation of chlorfenvinphos with great efficiency.

### 3.7. Effect of Oxygen

**Figure 10:**
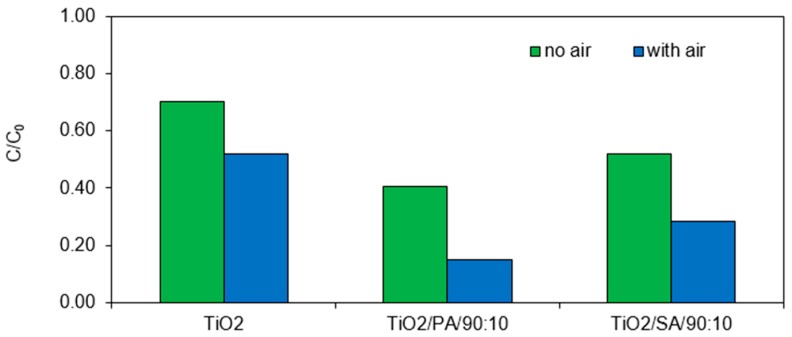
The efficiency of the chlorfenvinphos photodegradation in the presence of selected photocatalysts, with and without additional aeration. Conditions of experiment: C_0_ = 1.0 mg/L; D_c_ = 50.0 mg/L; V_r_ = 500.0 mL; pH = 6.0; t_sor_ = 20.0 min; t_ir_ = 60.0 min.

A comparative assessment was carried out for the chlorfenvinphos photocatalysis process with and without oxygen. Based on the results presented in Figure 10 it can be concluded that the presence of dissolved oxygen in the reaction mixture plays a significant role in the process of removing chlorfenvinphos. It should be emphasized that the results presented in earlier sections concerned the processes without the oxygen in the reaction system. After 60 min of the process, the CFVP decomposition was about 27%, 63%, and 48% for pure TiO_2_, TiO_2_/PA/90:10 and TiO_2_/SA/90:10 respectively. Conducting the process of chlorfenvinphos decomposition with aeration resulted in an increase in the efficiency of the degradation process by about 20%. The increase in the efficiency of reactions carried out in the presence of oxygen was also confirmed on the base of reaction kinetics analysis (Table 6). Oxygen introduced into the reaction system contributes to a more effective decomposition of CFVP, reducing the half-life from 257 min to 98 min in the presence of the pure titanium (IV) oxide. The mathematically calculated half-life of CFVP has been reduced by about two times in the presence of the modified photocatalysts. The reason for this phenomenon is adsorption of oxygen on the catalysts surface. Oxygen is an electron acceptor and prevents the recombination of the electron-hole pairs. In addition, oxygen participates in the oxidation reaction of the chlorfenvinphos and intermediate products formed during the reaction [49,50].

### 3.8. Radical Scavengers Test

**Figure 11:**
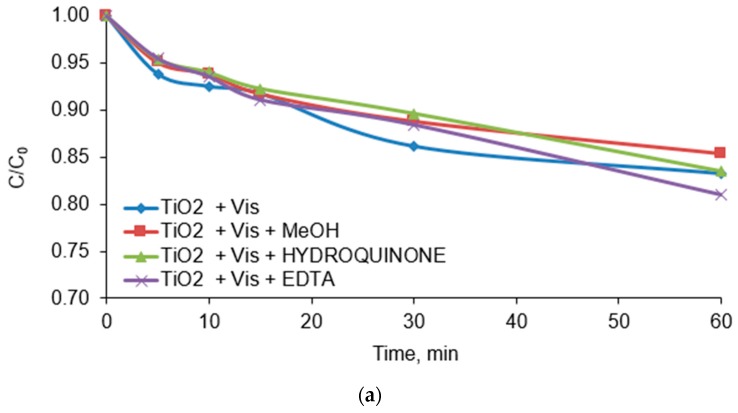

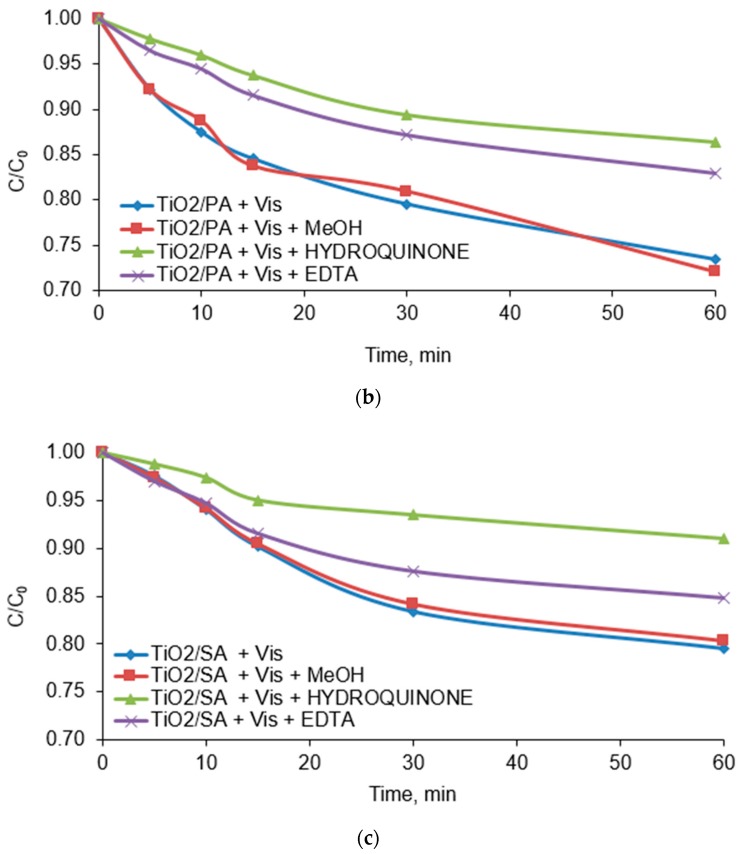
Photodegradation of the CFVP catalysed by (**a**) TiO_2_, (**b**) TiO_2_/PA/90:10 and (**c**) TiO_2_/SA/90:10 in the presence of radical scavengers. Conditions of experiment: C_0_ = 1.0 mg/L; D_c_ = 50.0 mg/L; V_r_ = 500.0 mL; pH = 6.0; min; t_ir_ = 60.0 min, D_scav._ = 50.0 µM.

In order to identify the main active species involved in the CFVP photodegradation, tests were carried out. Methanol, hydroquinone and EDTA-2Na were used in the experiments as OH^•^ scavengers, O_2_^•−^ scavengers and h^+^ holes scavengers respectively. These compounds are commonly used as radical scavengers by the authors of many papers [51,52,53,54,55]. The scavengers were always used in the concentration of 50.0 µM. The effect of individual scavengers is shown in Figure 11. In general, as a result of irradiating solutions with the Vis light, the degree of the CFVP degradation decreased after the addition of the O_2_^•−^ scavenger (hydroquinone) and the hole scavenger (EDTA-2Na) (Figure 10a). On the other hand, the addition of the OH^•^ radical scavenger did not reduce the effectiveness of pesticide degradation. In the presence of pure titanium (IV) oxide, the reduction of CFVP concentration (in the range from 15 to 19%) was mainly due to the adsorption of the pesticide by the adsorbent (TiO_2_). In the TiO_2_/Vis system, the inhibition of the chlorfenvinphos decomposition by the addition of methanol is due to the fact that it is partially activated by visible radiation, however, if solar radiation is used, only 3–5% of the energy can be used for the TiO_2_ activation [56]. The addition of hydroquinone and EDTA-2Na to the TiO_2_/VIS system did not cause significant inhibition of the process. As shown in Figure 10b,c, addition of O_2_^•−^ and h^+^ scavengers in the systems of TiO_2_/PA and TiO_2_/SA contributed to significant inhibition of the CFVP decomposition process. For example, in the system of TiO_2_/PA/90:10 the pesticide degradation rate after adding hydroquinone has decreased from *k* = 0.046 min^−1^ (control) to *k* = 0.0024 min^−1^ (Table 7). The half-life of chlorfenvinphos under these conditions has lengthened from 151.0 min to 289.0 min and 231.0 min after adding hydroquinone and EDTA-2Na respectively. Similar effects were observed for theTiO_2_/SA/90:10. The reaction rate constant of the control sample decreased from *k* = 0.039 min^−1^ (*t/2* = 462.0 min) to *k* = 0.026 min^−1^ (*t/2* = 267.0 min) after adding EDTA-2Na. The addition of methanol as an OH^•^ inhibitor does not significantly reduce the rate of CFVP degradation. Therefore it can be concluded that in the photodegradation process of the tested pesticide, the most active radical is O_2_^•−^, then h^+^ and least OH^•^.

The dominance of O_2_^•−^ radicals may result from the reduction in the size of the band gap energy of the synthesized catalysts. The values of the band gap energy and the location of valence and conduction bands were shown in Section 3.1 (Figure 4). Due to the reduction of the band gap energy of the modified photocatalysts, the transfer of electrons from the valence band (VB) to the conductivity band (CB) is facilitated. Along with the transfer of electron, an electron hole (h^+^) is created, i.e., an unoccupied energy level that is involved in the photooxidation processes of pollutants adsorbed on the surface of the TiO_2_/PA and TiO_2_/SA photocatalysts. The more electrons (e^−^) go into the conductivity band, the more O_2_^•−^ radicals are generated.

## 4. Summary

A TiO_2_/PA and TiO_2_/SA photocatalysts with visible-light photocatalytic activity in the chlorfenvinphos pesticide degradation was synthesized. It was found that the pyruvic and succinic acid can be used for modification of the commercial TiO_2_ and improvement of its photocatalytic activity.

Modification of the commercial titanium (IV) oxide with organic acids allowed to decompose the pesticide under the influence of visible light. The effect of photocatalysts dose on the chlorfenvinphos decomposition efficiency was found. Too low dose contributed to the unsatisfactory parameters of the compound decomposition. The high dose caused too fast sedimentation of the catalysts and their photooxidizing potential was not used. The dose of 50 mg/L was chosen as the optimal dose of the photocatalysts.

The use of modification extended the life of the catalysts with high photodegradation efficiency. Moreover, in the presence of dissolved oxygen the degree of the compound degradation increased by approximately 20%. However, the oxidation processes of chlorfenvinphos, catalysed by the modified TiO_2_, were more effective even without the introduction of an additional source of oxygen.

Due to the surface charge of the catalysts, the processes carried out in the presence of the commercial TiO_2_ were most effective at pH close to the isoelectric point value (pH_pzc_), i.e., at the pH = 6.0. In turn, the value of the pH_pzc_ point of the modified photocatalysts probably changed, because at low values (pH = 3.0) the chlorfenvinphos photodegradation was the most effective.

The dominant species involved in the CFVP photodegradation process were peroxide radicals and electron holes. The hydroxyl radicals were least involved in the CFVP decomposition process.

The photodegradation process conducted in the presence of the modified composites increases the rate of the chlorfenvinphos degradation, as indicated by the analysis of the pseudo first-order parameters. The half-life of the tested compound was on average three times lower than in the case of solutions containing the pure TiO_2_.

According to the presented results, higher photocatalytic activity of the TiO_2_/PA and TiO_2_/SA can be attributed to: reducing the phenomenon of the TiO_2_ agglomerate formation, which in turn led to the increase in size of the active surface, and thus TiO_2_ activity (a); increase in specific surface area of the modified photocatalysts, thus increasing the adsorption capacity (b); inhibition of transformation of anatase into rutile by eliminating formation of the structural defects during modified catalysts preparation (c); slowing down the recombination process of electron-hole pairs through the separation of excited charges (d); reduction of the band gap energy relative to the pure titanium dioxide (e). The photooxidation of chlorfenvinphos under the influence of visible light could also be possible as a result of photosensibilization of the commercial TiO_2_ due to the transfer of electrons from PA/SA to the TiO_2_ conductivity band and appearing of a new C-O-Ti bond (shift of the absorption band towards longer waves λ > 400 nm), where the elemental carbon (C) comes from the pyruvic or succinic acid [18,19,57,58].

## Figures and Tables

**Table 1:** Physico-chemical characteristics of chlorfenvinphos.

Chemical Structure	Physico-Chemical Properties	
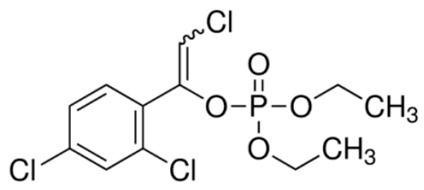	Molecular formula	C12H14Cl3O4P
	Molecular weight (g/mol)	359.57
	CAS number	470-90-6
	Water solubility at 20 °C (mg/L)	124.0
	Density (g/L)	1.36
	Vapor pressure (25 °C) (mmHg)	7.5 × 10^−6^
	logKOW (−)	3.81

**Table 2:** Physico-chemical characteristics of TiO_2_.

Chemical Structure	Physico-Chemical Properties	
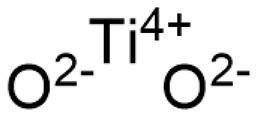	Symbol, origin	P-25, Sigma-Aldrich (Poznań, Poland)
	Crystal structure	Anatase:rutile = 80:20
	Surface area (m^2^/g)	35.0–65.0
	Particle size (nm)	21.0
	Density (g/cm^3^)	4.26

**Table 3:** Symbols of the photocatalysts.

Symbol	Explanation
TiO_2_	Pure Titanium(IV) Oxide
TiO_2_/PA/99:1	TiO_2_ modified with pyruvic acid in a ratio of 99:1 (*w*/*w*)
TiO_2_/PA/90:10	TiO_2_ modified with pyruvic acid in a ratio of 90:10 (*w*/*w*)
TiO_2_/PA/80:20	TiO_2_ modified with pyruvic acid in a ratio of 80:20 (*w*/*w*)
TiO_2_/PA/50:50	TiO_2_ modified with pyruvic acid in a ratio of 50:50 (*w*/*w*)
TiO_2_/PA/20:80	TiO_2_ modified with pyruvic acid in a ratio of 20:80 (*w*/*w*)
TiO_2_/SA/99:1	TiO_2_ modified with succinic acid in a ratio of 99:1 (*w*/*w*)
TiO_2_/SA/90:10	TiO_2_ modified with succinic acid in a ratio of 90:10 (*w*/*w*)
TiO_2_/SA/80:20	TiO_2_ modified with succinic acid in a ratio of 80:20 (*w*/*w*)
TiO_2_/SA/50:50	TiO_2_ modified with succinic acid in a ratio of 50:50 (*w*/*w*)
TiO_2_/SA/20:80	TiO_2_ modified with succinic acid in a ratio of 20:80 (*w*/*w*)

**Table 4:** Impact of the TiO_2_ modification on the pseudo first-order parameters.

TiO_2_/PA				TiO_2_/SA			
Symbol	*k*, 1/min	*R* ^2^	*t/2*, min	Symbol	*k*, 1/min	*R* ^2^	*t/2*, min
TiO_2_	0.0027	0.86	257.0	TiO_2_	0.0027	0.86	257.0
99:1	0.0046	0.87	151.0	99:1	0.0034	0.94	204.0
90:10	0.0110	0.93	63.0	90:10	0.0064	0.93	108.0
80:20	0.0063	0.93	110.0	80:20	0.0039	0.91	178.0
50:50	0.0036	0.81	193.0	50:50	0.0031	0.86	224.0
20:80	0.0022	0.91	315.0	20:80	0.0031	0.80	224.0

**Table 5:** Effect of pH on the kinetics of the process.

pH	Catalyst	*k*, 1/min	*R* ^2^	*t/2*, min
3	TiO_2_	0.0019	0.85	365.0
	TiO_2_/PA/90:10	0.0104	0.97	67.0
	TiO_2_/SA/90:10	0.0063	0.95	110.0
6	TiO_2_	0.0031	0.94	224.0
	TiO_2_/PA/90:10	0.0084	0.91	83.0
	TiO_2_/SA/90:10	0.0082	0.98	85.0
9	TiO_2_	0.0027	0.82	257.0
	TiO_2_/PA/90:10	0.0057	0.82	122.0
	TiO_2_/SA/90:10	0.0078	0.86	89.0

**Table 6:** Effect of aeration of systems on the process kinetics.

Without Aeration				With Aeration			
Symbol	*k*, 1/min	*R* ^2^	*t/2*, min	Symbol	*k*, 1/min	*R* ^2^	*t/2*, min
TiO_2_	0.0031	0.94	224.0	TiO_2_	0.0071	0.92	98.0
TiO_2_/PA/90:10	0.0084	0.91	83.0	TiO_2_/PA/90:10	0.0255	0.97	27.0
TiO_2_/SA/90:10	0.0082	0.98	85.0	TiO_2_/SA/90:10	0.0178	0.93	39.0

**Table 7:** Effect of radical scavengers on photodegradation kinetics.

Catalyst	Scavenger	*k*, 1/min	*R* ^2^	*t/2*, min
TiO_2_	Control	0.0027 *	0.86	257.0
	MeOH	0.0023	0.87	301.0
	Hydroquinone	0.0027	0.94	257.0
	EDTA	0.0032	0.95	217.0
TiO_2_/PA/90:10	Control	0.0046	0.87	151.0
	MeOH	0.0049	0.90	141.0
	Hydroquinone	0.0024	0.92	289.0
	EDTA	0.0030	0.92	231.0
TiO_2_/SA/90:10	Control	0.0039	0.91	178.0
	MeOH	0.0037	0.91	187.0
	Hydroquinone	0.0015	0.90	462.0
	EDTA	0.0026	0.88	267.0

* The difference between values *k*, *R*^2^, and *t/2* presented in Section 3.5 and Section 3.6 and the values in the Table above results from the fact that the CFVP adsorption is included in the mentioned sections.
